# Will Coiling Survive through the Next Decade?

**DOI:** 10.3390/jcm11113230

**Published:** 2022-06-06

**Authors:** Hans Henkes, Joachim Klisch, Pedro Lylyk

**Affiliations:** 1 Neuroradiologische Klinik, Klinikum Stuttgart, Kriegsbergstrasse 60, D-70174 Stuttgart, Germany; 2 Diagnostische und Interventionelle Radiologie und Neuroradiologie, Helios Klinikum Erfurt, Nordhäuser Str. 74, D-99089 Erfurt, Germany; joachim.klisch@helios-gesundheit.de; 3 Clinica La Sagrada Familia, Av. del Libertador 6647, Buenos Aires C1428 CA, Argentina; pdalldorf@lylyk.com.ar

## 1. Introduction

During the past three decades, neuroendovascular therapy has evolved from a focus on new disease concepts to revised treatment strategies and, ultimately, to versatile new technologies. Important technological developments in all fields typically pass through several revolutionary periods, which are then followed by phases of consolidation [1]. The two most significant technological innovations developed for the endovascular treatment of intracranial aneurysms are controlled coil detachment [2,3] and flow diversion [4].

Coil technology has undergone spectacular development since its inception [5]. Once electrothrombosis was disproven as its underlying mechanism of action [6], alternative detachment modes were developed to facilitate more rapid coil release than could be achieved with electrolysis alone [7,8]. The clinical availability of three-dimensional and extremely soft finishing coils, as well as the development of hydrocoils (MicroVention) all contributed to the consolidation of coil technology [9,10].

However, coil occlusion now joins detachable balloons, embolization with spheres, and the use of particles and liquid agents as an “old” neuroendovascular technique. Consequently, a question arises as to whether coiling modalities will survive through the next decade (i.e., 2022–2031).

Despite the ongoing rapid development and deployment of new (and newer) technologies, we believe that the answer to this question is yes. In this Editorial, we outline our arguments that support this viewpoint.

## 2. Our Institutional Perspectives

A preliminary answer to this question might emerge from an understanding of the changes involved in the use of this device over time. A review of our collective experience during the past ten years of clinical practice will be used as the basis for this discussion.

Our perspectives are based on our collective experience with aneurysm treatment in our clinical practices at Klinikum Stuttgart (HH), Helios Klinikum Erfurt (JK), and Clinica La Sagrada Familia, ENERI, Buenos Aires (PL) (Table 1).

The primary catchment area of Klinikum Stuttgart includes approximately two million individuals. Two other hospital departments are capable of providing endovascular treatment of intracranial aneurysms to some, if not all, the inhabitants in this region. By contrast, Klinikum Erfurt is a single institution and the sole provider of endovascular therapy for approximately 300,000 inhabitants. Clinica La Sagrada Familia has a primary catchment area that includes 13 million people (Ciudad Autonoma de Buenos Aires and Gran Buenos Aires). In this area, there are an additional 15 institutions capable of providing endovascular aneurysm treatment. Each of these three departments receives a significant number of referrals from individuals living in regions that are beyond their catchment areas.

The three institutions that contributed to this Editorial have the following characteristics:

- They offer a full range of neurovascular services.
- They can also provide microsurgical treatments.
- They are equipped with biplane digital subtraction angiography (DSA) systems.
- They have access to all certified access products and implants.
- They specialize in the treatment of aneurysms.
- They are asked to treat a variety of straightforward and challenging cases.
- They can offer endovascular treatment to nearly all aneurysm patients.

Patients are referred to these centres if endovascular treatment is considered to be one of the potential therapeutic options. Conversion from an attempted endovascular to a microsurgical procedure occurs only rarely. The differences in device usage reflect the personal preferences of the authors.

At the Stuttgart clinic, the caseload of patients diagnosed with a cerebral aneurysm increased from 2011 to 2019. Fewer patients with aneurysms were treated in 2020 because of the COVID-19 pandemic. Coil use decreased substantially during this interval. While 1557 units were used in 2014, only 928 were used in 2019. However, the comparatively large number of stent-assisted coiling procedures mainly reflects the use of bifurcation stents. The number of Woven EndoBridge (WEB) procedures was and remains small due to team preferences. Beginning in 2013, more aneurysms were treated with flow diverters than coils. The use of flow diverters fluctuated for various reasons, reaching a peak value of 393 units in 2015. A comparatively large number of extrasaccular flow diversion procedures were performed. This is a reflection of the departments’ clinical and scientific focus.

The Erfurt clinic used many more WEB devices than the other sites. The use of extrasaccular flow diverters at this clinic increased steadily since 2011, from 12 to 46 per year, while coil use varied between 500 and 700 per year since 2011 without any evidence of a steady decline. While the aneurysm caseload at this site increased from 2011 to 2018, a slight decrease was noted in 2020. Since 2012, a constant fraction (approximately 30%) of the aneurysms presenting at this facility were treated with a WEB device.

The results reported from the Buenos Aires clinic revealed a predominance of coiling; a significant but uniformly smaller fraction of aneurysms was treated with flow diversion. The reduced number of procedures performed in 2020 can again be explained by the impact of the COVID-19 pandemic.

Fewer WEB devices were used by both the Stuttgart and Buenos Aires clinics when compared to the Erfurt report (i.e., Stuttgart, *n* = 8; Erfurt, *n* = 278; Buenos Aires, *n* = 49 for the years 2011–2020). The number of coils per aneurysm is routinely between four and eight; the average number of coils used per procedure was lower in the Erfurt series compared to the other two clinics.

In general, the case-load (extraordinary large in Buenos Aires), the proportion of flow diverter procedures (relatively high in Stuttgart), the number of WEB implantations (with numbers in Erfurt exceeding those in Stuttgart and Buenos Aires together), and the frequent use of balloon remodelling (in Buenos Aires) represent, at least to some extent, unique characteristic features of each institution’s practice. The three contributing institutions share a fluctuating, but not consistently declining number of coils used on an annual basis.

In summary, the institutional caseloads and treatment strategies used by each of these three specialty clinics differed significantly. We assumed that this variation reflects global differences. Although new treatment strategies (e.g., extrasaccular flow diversion and, to a much lesser extent, intrasaccular flow disruption) have gained a substantial market share, coil use remains robust. The increase in the use of extrasaccular flow diversion procedures during the past decade was much more prominent than the increases observed in intrasaccular flow disruption (e.g., WEB). This may be due in part to the fact that WEB was introduced somewhat later on during the decade.

## 3. What Are the Goals of Endovascular Aneurysm Treatment, and to What Extent Are They Achieved?

For most patients, the goal of endovascular treatment is to prevent aneurysmal haemorrhage or re-haemorrhage without procedure-related morbidity or mortality.

Early re-rupture (i.e., during Post-procedure Days 1–3) occurs in 1–3.6% of the cases in which coil occlusion is used to treat ruptured aneurysms [11]. The rate of late bleeding or rebleeding after coil occlusion does not exceed 2% [12,13,14]. Partial coil occlusion, coil migration into an intrasaccular thrombus, or coil compaction can result in aneurysm reperfusion and potential (re-)rupture. Aneurysms that develop these post-procedural complications need stringent follow-up and frequently require retreatment.

Intracranial haemorrhage after extrasaccular flow diversion remains a concern, particularly when treating blister aneurysms [15] and aneurysms with a fundus diameter ≥10 mm [16,17]. The rate of complete aneurysm occlusion within one year is >80% with combined morbidity and mortality <5% [18]. Aneurysm recurrence and (re-)bleeding after complete occlusion secondary to flow diversion are exceedingly rare.

By contrast, the long-term results associated with WEB devices have not yet been clarified, and the risk of late (re-)rupture after WEB-based occlusion procedures remains unknown. The risks associated with the procedure are low, with 4% morbidity and 1% mortality [19]. However, ~20% of WEB-occluded aneurysms may need a second treatment [20].

## 4. The Regional and Global Perspective

**Figure 1:**
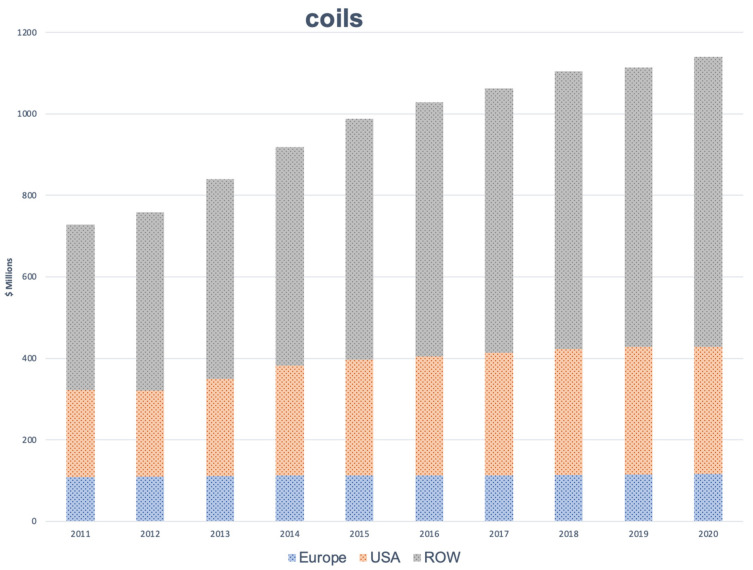
Annual revenues (in millions USD) associated with the use of endovascular coils in Europe, the USA, the rest of the world (ROW), and globally (totals) from 2011 through 2020. Data were collected from industry sources.

It is important to consider these findings from the perspective of the revenues associated with different products and their markets worldwide. Revenues associated with endovascular coil-based procedures increased moderately in Europe between 2011 and 2020, increasing from USD 81 to 84 million per year. In the United States (USA), revenues have increased from USD 213 to 313 million per year. Increased revenues were also reported in the rest of the world (ROW; USD 406 to 711 million per year) (Figure 1).

In 2020, the global revenues associated with the use of endovascular coils were USD 1140 million; these revenues are expected to increase to USD 1175 million during the year 2021, despite the obstacles presented by the COVID-19 pandemic.

**Figure 2:**
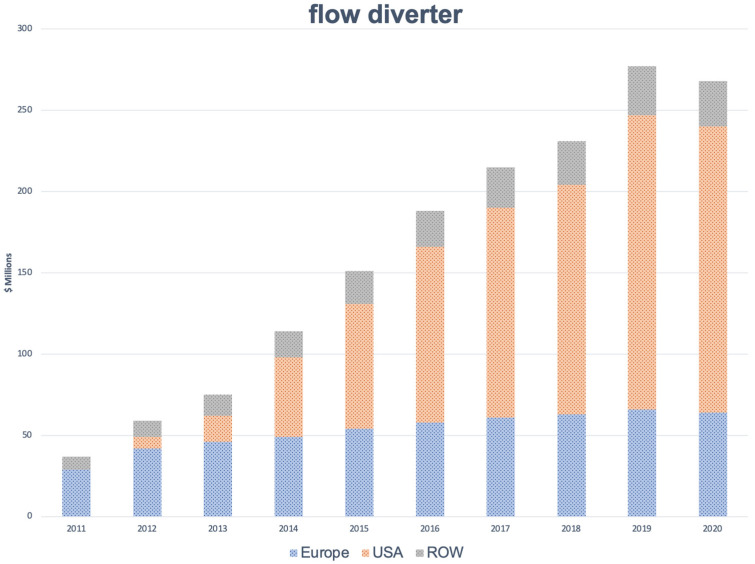
Annual revenues (in millions USD) associated with the use of flow diverters in Europe, the USA, the rest of the world (ROW), and globally (total) from 2011 through 2020. Data were collected from industry sources.

During the same period (2011–2020), revenues associated with the use of flow diverter devices (and corresponding product unit sales) increased from USD 29 to 64 million per year in Europe (i.e., 2600 to 6350 units). In 2012, the market for flow diverter devices in the USA was USD 7 million and grew to USD 176 million in 2020 (representing 560 and 12,900 flow diverter devices, respectively). The corresponding values for the ROW were comparatively modest due to the slow regulatory processes in several critical markets (Figure 2).

Since their introduction in Europe in 2008 and current broad use worldwide, flow diverters have not replaced coils in any market. By contrast, coiling remains the preferred approach for certain types of aneurysms (e.g., giant aneurysms, partially thrombosed aneurysms) to avoid delayed rupture. Implants that disrupt intrasaccular flow are particularly useful and can replace coils in cerebral aneurysm therapy. Geographic differences also appear to be driving changes in coil sales. For reasons unclear, the MEDINA device (Medtronic) was taken off the market [21], while LUNA and ARTISSE (both from Medtronic) have not yet been launched [22]. WEB (MicroVention) implants received U.S. Food and Drug Administration (FDA) approval in the spring of 2019 and are currently the targets of substantial interest [23]. Since its introduction in 2010 [24], the WEB device has continually undergone product improvements and updates; the most recent version is known as WEB 17. WEB devices have been evaluated in several clinical trials and are currently among the best-studied of all neuroendovascular implants used in clinical practice. According to the experience of one author (J.K.), 25–30% of all aneurysms may be suitable for WEB treatment. Although WEB implantation has resulted in some disappointment among those hoping to avoid aneurysm reperfusion and retreatment rates in the range of 20% have been reported [20], occlusion rates may be higher than those achieved with bare coiling alone. The main advantage of WEB implants lies in their potential to occlude wide-necked aneurysms without the use of a stent. This modality may then be suitable for treating acutely ruptured aneurysms, as dual antiplatelet therapy may not be needed in the absence of a stent. In 2020, MicroVention reported the treatment of >12,000 aneurysms with their WEB system; this is a sobering number, given the 10-year history of this device. Contour (Cerus) is in some ways a simplified variation of a WEB that shares similar indications and limitations. The Contour version appears to be easier to use, and its use might eventually surpass both WEB implants and coils [25]. However, since their introduction to the market in 2010, intrasaccular flow disruptors have neither replaced coils nor significantly decreased their sales.

New products that provide improved access (e.g., steerable microcatheters and improved guidewires) might be effective at driving coil usage. Expanding indications (e.g., the ability to treat unruptured aneurysms) may have a similar effect. However, in the future, stent-assisted coiling may be limited to bifurcation aneurysms, because flow diversion already has the potential to replace coiling for those affecting the side walls.

However, at this time, it remains to be seen whether any device or method will replace the use of coils in current or future clinical practice. There are several arguments in favour of the use of coils. Among these points, coils are now affordable, easy to use, relatively neutral with respect to microcatheters, and available in numerous sizes and with a large variety of physical features (stiffness and shape). The average prices of coils, flow diverters, adjunctive, and access products for aneurysm treatment in 2020 are shown in Table 2.

Coils can be combined with other implants and used to treat both ruptured and unruptured aneurysms without the need for any specific adjunct medication. Coils are the mainstay of several other neuroendovascular embolization procedures, including dural arteriovenous fistulas (DAVFs), fistulous arterio-venous malformation (AVMs), and parent vessel occlusion. Thus, coils are more versatile than other devices, which are limited to the treatment of aneurysms. While accurate stepwise filling of an aneurysm with coils is a conceptually different modality (and substantially more time-consuming) than “one-and-done” WEB implantation, each has advantages and drawbacks. The main disadvantage of coils is the comparatively high rate of aneurysm recurrence, despite the low rate of post-procedural bleeding.

Flow diverters are expensive, are not always suitable for bifurcation aneurysms, and are currently not widely used to treat aneurysms that have ruptured. Flow diverters still require adjunctive coiling in certain situations, and at least mono- if not dual antiplatelet therapy remains necessary. By contrast, flow diversion has the lowest recurrence rate among all endovascular aneurysm treatment modalities.

The WEB system is also expensive and more complex to use than coiling or extrasaccular flow diversion, and aneurysms can recur post-treatment. The main indications for WEB are broad-based bifurcation aneurysms. In these cases, WEB implants can be used to simplify what would otherwise be challenging procedures (e.g., wide-necked basilar bifurcation aneurysms in the acute phase after rupture). The largest available WEB implant has a diameter of 11 mm and requires a microcatheter with a 3.4 F distal outer diameter. Both features result in limitations for the clinical usage of this device.

**Figure 3:**
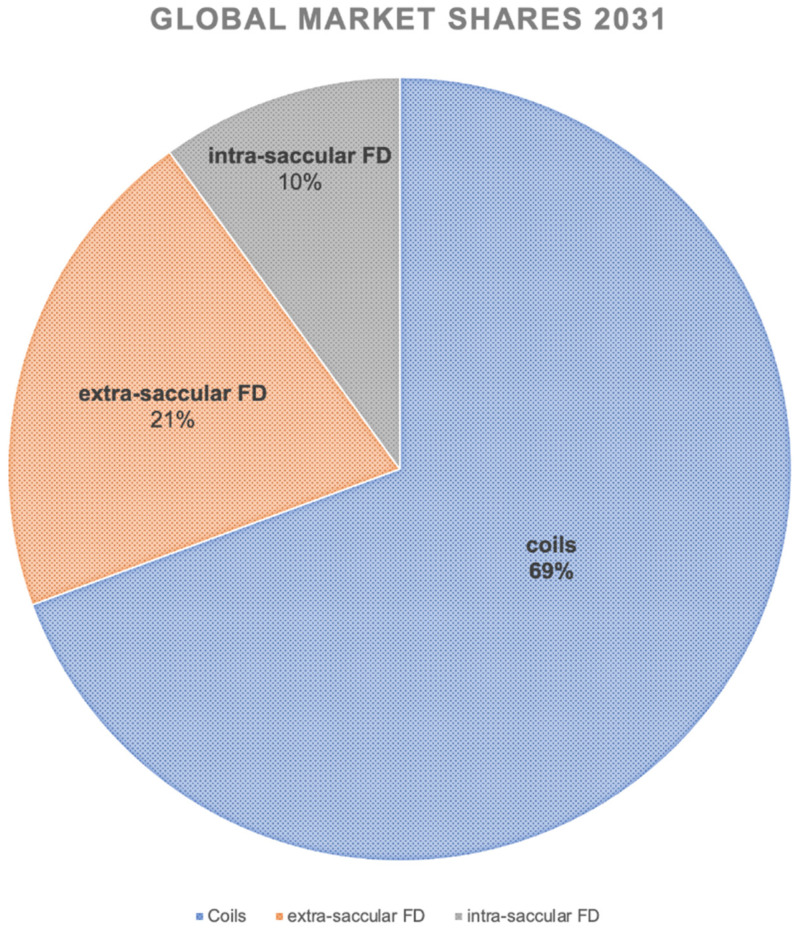
Expected global market shares of aneurysm treatment products by the year 2031. These data were extrapolated from the development of the market shares during the last decade.

In 2020, the global market shares were divided into 85% coils vs. 15% for extrasaccular flow diverters, with a limited impact from WEB and similar devices. Industry experts anticipate that, in the year 2025, the division will be 70%, 20%, and 10% for coils, extrasaccular, and endosaccular flow diverters. Extrapolating to the year 2031, we might expect to see the following global market shares of aneurysm treatment products: 65–70% coils, 20–25% extrasaccular flow diverters, and 10% intrasaccular flow diverters (Figure 3).

## 5. Conclusions

At least for now, coiling is here to stay.

## Figures and Tables

**Table 1:** Number of aneurysms treated with coil occlusion, flow diversion, or WEB implantation and the annual use of coil and flow diverter (FD) units during the years 2011–2020 in three hospital-based specialized neuroendovascular departments, Klinikum Stuttgart (Stuttgart, Germany), Helios Klinikum Erfurt (Erfurt, Germany), and Clinical La Sagrada Familia, ENERI (Buenos Aires, Argentina); ^1^ values for coiled aneurysms only; ^2^ coil occlusion procedures performed to treat aneurysms and for all other indications; ^3^ annual consumption of detachable coils for any indication; ^4^ the mean number of coils used per aneurysm; ^5^ the number of aneurysm treatments that included the use of a self-expanding stent deployed to support coil occlusion; ^6^ the number of aneurysm treatments that included the use of a remodelling balloon catheter to support the coil occlusion; ^7^ the number of aneurysm treatments that included the deployment of a flow diverter stent; ^8^ the annual consumption of flow diverter stents; ^9^ the number of WEB devices implanted (typically one per aneurysm).

Klinikum Stuttgart	2011	2012	2013	2014	2015	2016	2017	2018	2019	2020
Ruptured/unruptured coiled aneurysms ^1^	44 65	56 89	51 89	54 110	42 89	61 92	45 78	67 97	44 84	50 42
Coil occlusion procedures ^2^	146	181	167	182	157	164	153	190	156	126
Coil units used ^3^	1290	1585	1209	1557	1199	1068	1346	1282	928	1060
Coils per aneurysm ^4^	7.5	7.6	7.4	7.6	6.5	5.7	7.3	6.5	6.4	7.4
Stent-assisted coiling ^5^	22	27	36	43	32	35	36	47	29	16
Balloon-assisted coiling ^6^	5	5	8	9	9	2	2	9	9	4
Flow diversion (FD) procedures ^7^	112	124	146	197	226	209	185	239	227	186
FD units used ^8^	243	195	212	392	393	284	243	310	304	254
**Klinikum Erfurt**	**2011**	**2012**	**2013**	**2014**	**2015**	**2016**	**2017**	**2018**	**2019**	**2020**
Ruptured/unruptured coiled aneurysms ^1^	29 41	34 30	31 29	34 29	19 12	27 20	30 22	29 21	36 19	24 8
Coil occlusion procedures ^2^	80	80	78	73	50	66	65	72	74	48
Coil units used ^3^	562	595	593	558	468	478	595	632	691	512
Coils per aneurysm ^4^	5	5.7	5.9	5.8	5.5	6.4	6.4	5.1	5.1	4.2
Stent-assisted coiling ^5^	15	16	12	17	10	4	8	10	9	10
Balloon-assisted coiling ^6^	0	0	0	0	2	1	0	2	1	0
FD procedures ^7^	12	9	12	20	15	10	28	35	42	46
FD units used ^8^	13	9	13	20	15	12	26	35	41	47
Woven EndoBridge (WEB) procedures = units ^9^	7	21	29	33	36	31	33	37	27	24
**ENERI, Buenos Aires**	**2011**	**2012**	**2013**	**2014**	**2015**	**2016**	**2017**	**2018**	**2019**	**2020**
Ruptured/unruptured coiled aneurysms ^1^	182 273	204 303	211 318	223 330	196 319	228 349	199 330	247 359	228 575	133 279
Coil occlusion procedures ^2^	301	348	310	354	307	410	313	444	554	321
Coil units used ^3^	2107	2088	2170	2407	2456	3034	2253	3063	4055	2195
Coils per aneurysm ^4^	7	6	7	6.8	8	7.4	7.2	6.9	7.3	6.8
Stent-assisted coiling ^5^	40	64	22	29	33	66	62	59	68	48
Balloon-assisted coiling ^6^	80	91	90	80	42	62	66	67	71	95
FD procedures ^7^	154	148	204	186	199	166	216	160	249	91
FD units used ^8^	187	214	213	229	218	213	236	203	274	102
WEB procedures = used units ^9^	0	11	15	13	9	1	0	0	0	0

**Table 2:** Average price (or price range; in €) of medical devices used for the endovascular treatment of intracranial aneurysms in Germany and Argentina in 2020. Costs are based on information from various industry sources; balloon, remodelling balloon catheter; FD, flow diverter; WEB, Woven EndoBridge).

**Product**	Coil	FD	WEB	Self-Expanding Stent
Germany	290	9500	9950	2800
Argentina	580	6700	6200	3600
**Product**	Balloon	Guide Catheter	Microcatheter	Microwire
Germany	600	90–1000 **	330	220
Argentina	110 *–2200	440–900 **	800	350

* Most physicians use coronary balloon catheters for remodelling; ** triaxial access has become common practice.

## Data Availability

There is no data beyond the content of this manuscript.
